# Morphological and Transcriptional Responses to CRISPRi Knockdown of Essential Genes in Escherichia coli

**DOI:** 10.1128/mBio.02561-21

**Published:** 2021-10-12

**Authors:** Melanie R. Silvis, Manohary Rajendram, Handuo Shi, Hendrik Osadnik, Andrew N. Gray, Spencer Cesar, Jason M. Peters, Cameron C. Hearne, Parth Kumar, Horia Todor, Kerwyn Casey Huang, Carol A. Gross

**Affiliations:** a Department of Cell and Tissue Biology, University of California San Francisco, San Francisco, California, USA; b Department of Bioengineering, Stanford University, Stanford, California, USA; c Department of Microbiology and Immunology, Stanford University School of Medicine, Stanford, California, USA; d Pharmaceutical Sciences Division, School of Pharmacy, University of Wisconsin—Madison, Madison, Wisconsin, USA; e Chan Zuckerberg Biohub, San Francisco, California, USA; National Cancer Institute

**Keywords:** arrayed library, CRISPRi, *Escherichia coli*, essential genes, microscopy

## Abstract

CRISPR interference (CRISPRi) has facilitated the study of essential genes in diverse organisms using both high-throughput and targeted approaches. Despite the promise of this technique, no comprehensive arrayed CRISPRi library targeting essential genes exists for the model bacterium Escherichia coli, or for any Gram-negative species. Here, we built and characterized such a library. Each of the ∼500 strains in our E. coli library contains an inducible, chromosomally integrated single guide RNA (sgRNA) targeting an essential (or selected nonessential) gene and can be mated with a pseudo-Hfr donor strain carrying a *dcas9* cassette to create a CRISPRi knockdown strain. Using this system, we built an arrayed library of CRISPRi strains and performed population and single-cell growth and morphology measurements as well as targeted follow-up experiments. These studies found that inhibiting translation causes an extended lag phase, identified new modulators of cell morphology, and revealed that the morphogene *mreB* is subject to transcriptional feedback regulation, which is critical for the maintenance of morphology. Our findings highlight canonical and noncanonical roles for essential genes in numerous aspects of cellular homeostasis.

## INTRODUCTION

Reverse genetic approaches based on gene inactivation have been responsible for elucidating the function of many bacterial genes (1–4). Essential genes, which encode the key reactions of life and represent a large fraction of a cell’s protein budget (5), are not amenable to such approaches because, by definition, their deletion renders cells inviable. CRISPR interference (CRISPRi) provides inducible knockdown of bacterial gene expression (6, 7) and has enabled genetic approaches to studying essential gene function. Arrayed libraries of CRISPRi strains targeting essential genes have been of particular utility, because they enable flexible pooling and candidate follow-ups of pooled assays as well as single-strain assays such as microscopy. Such libraries have been described for Bacillus subtilis (8), Streptococcus pneumoniae (9), Streptococcus mutans (10), and Mycobacterium smegmatis (11) and have been used to yield surprising cross-pathway functional interactions, insights into cellular vulnerabilities, and functional characterizations of essential genes. Remarkably, no such library has been described for any Gram-negative bacterium despite the original demonstration of CRISPRi in Escherichia coli (7), the veritable cornucopia of pooled CRISPRi studies in Gram-negative bacteria (reviewed in reference 12), and the significant differences between Gram-negative and Gram-positive physiology centered around essential functions such as the Gram-negative-specific outer membrane.

Here, we describe the design and construction of such a library in E. coli. Motivated by the potential to uncover both fundamental and clinically relevant principles governing bacterial growth, we profiled the morphological and growth phenotypes of our library. We discovered increased lag upon knockdown of ribosomal genes, novel modulators of cell morphology, and a critical transcriptional feedback circuit affecting the expression of the cell shape gene *mreB*. This library, which is available from the Coli Genetic Stock Center, will be a resource for the microbiology community and will contribute to a deeper understanding of Gram-negative bacteria.

## RESULTS

### A partial arrayed library of chromosomally encoded CRISPRi strains.

**FIG 1:**
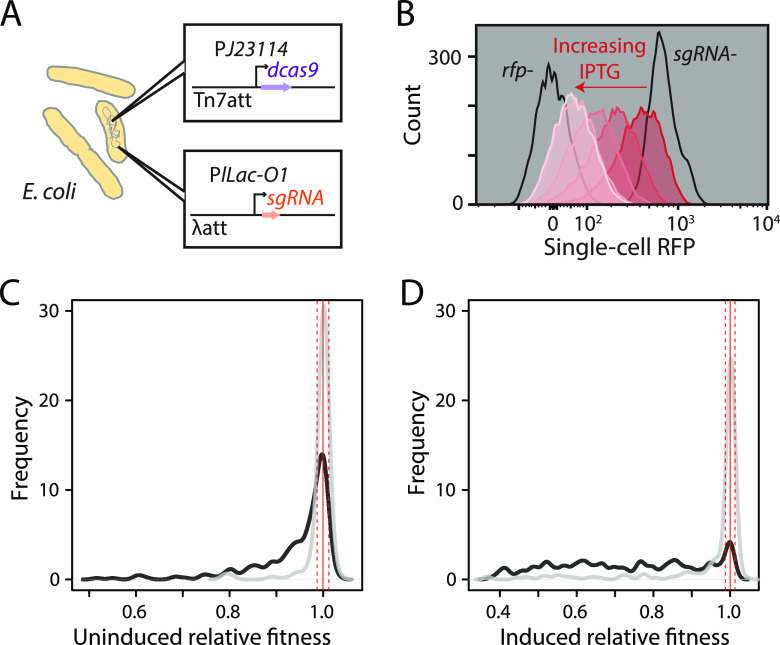
CRISPRi induction reduces fitness when targeting essential genes. (A) Schematic of the E. coli CRISPRi system showing the integration sites and promoters of the *sgRNA* and *dcas9* constructs. (B) CRISPRi targeting *rfp* expression achieved titratable and unimodal knockdown at various IPTG levels. The red histograms correspond to 0, 0.03125, 0.0625, 0.125, and 1 mM IPTG. No RFP (*rfp-*) or full RFP expression (*sgRNA-*) is indicated in black. (C and D) Gaussian kernel density estimate of the distribution of relative fitness values for strains targeting essential (black) and nonessential (gray) genes with the CRISPRi system at basal induction (C) and full induction using 1 mM IPTG (D). The solid red line indicates the median of 48 nontargeting controls, and the dashed red lines indicate ±3 standard deviations (SD) from the median, estimated from the median absolute distance.

Previous CRISPRi systems in E. coli (7, 13–18) used plasmids to express the single guide RNA (sgRNA) component of the CRISPRi system and were constructed and screened in pools. Although such libraries enable quantification of growth phenotypes, they are unsuitable for targeted assays such as single-strain profiling of morphology and growth dynamics, and the need to grow them under selection may complicate the determination of phenotypes such antibiotic sensitivities. To overcome these limitations, we constructed an arrayed library containing a chromosomally encoded CRISPRi system targeting the essential genes of E. coli (see Materials and Methods; also, see Table S1 in the supplemental material). Briefly, sgRNAs targeting the 5′ ends of all essential open reading frames (ORFs) and selected nonessential genes (Table S1) were integrated at the lambda *att* site of E. coli BW25113 under the control of an IPTG (isopropyl-β-d-thiogalactopyranoside)-inducible promoter (see Materials and Methods) as previously described (19). CRISPRi strains were constructed using a high-efficiency conjugation system to transfer *dcas9* from the chromosome of a pseudo-Hfr donor strain to the chromosomal Tn*7 att* site of the sgRNA-encoding recipient (Fig. 1A; Materials and Methods). We quantified the performance of our system by measuring its ability to repress *rfp* expression. Our CRISPRi system achieved ∼50% knockdown of *rfp* in the absence of inducer (due to basal expression of the sgRNA) and uniform, concentration-dependent knockdown of *rfp* upon sgRNA induction with IPTG (Fig. 1B; Materials and Methods).

10.1128/mBio.02561-21.6 TABLE S1 Strain and primer information for the E. coli CRISPRi library. Download Table S1, XLSX file, 0.4 MB. Copyright © 2021 Silvis et al. 2021 Silvis et al. https://creativecommons.org/licenses/by/4.0/ This content is distributed under the terms of the Creative Commons Attribution 4.0 International license.

The construction of our library (see Materials and Methods) differed from that of other arrayed libraries (8–11) in that sgRNAs were cloned, integrated, single-colony purified, and sequence verified (Fig. S1A; Table S1) prior to the introduction of *dcas9*. While this method limits the rise of suppressors during strain construction, errors during the additional handling required to introduce *dcas9* into the sgRNA strains unfortunately led to substantial cross-contamination of the CRISPRi strains. This cross-contamination affected ∼30% of our CRISPRi strains (contamination fraction of >10^−4^ based on deep sequencing of each strain) (Table S1; Fig. S1B) but not the sgRNA strains (Table S1; Fig. S1A). Unfortunately, the cross-contamination was not discovered until after data collection was completed. As such, we discuss only those results pertaining to the 372 uncontaminated CRISPRi strains (Table S1). These strains encompass all major essential processes (all Clusters of Orthologous Groups [COG], Gene Ontology [GO], and KEGG categories with more than 5 genes) and hence retain the potential to uncover systems-level physiological consequences of essential gene knockdown.

10.1128/mBio.02561-21.1 FIG S1 A substantial fraction of CRISPRi strains, but not sgRNA strains, are contaminated. Histograms depict the log_10_ of the fraction of sequencing reads mapped to the major contaminant in the sgRNA (A) and CRISPRi (B) strains. The red line in panel B depicts the cutoff used for including CRISPRi strain results in this study. The CRISPRi strains (B) exhibited a bimodal pattern of contamination, with ∼400 strains clustered around the median contamination level of 1.8 × 10^−5^, ∼50 strains exhibiting intermediate levels of contamination, and ∼100 strains exhibiting complete or almost complete contamination. The sgRNA strains (A) exhibited a drastically lower maximum major contaminant fraction of 3.6 × 10^−3^. Although the sgRNA strains exhibited a higher median major contaminant fraction (4.3 × 10^−4^) than the CRISPRi strains, this was likely due to technical artifacts, as the major contaminant observed in 501/519 strains was *rplS*, which never occurred as a major contaminant in the CRISPRi strains (Table S1). Download FIG S1, PDF file, 0.07 MB. Copyright © 2021 Silvis et al. 2021 Silvis et al. https://creativecommons.org/licenses/by/4.0/ This content is distributed under the terms of the Creative Commons Attribution 4.0 International license.

### Pooled growth of the CRISPRi library illustrates essential gene dosage effects.

To determine the effect of essential gene knockdown using our system, we constructed a pooled version of our library (see Materials and Methods) and quantified the relative fitness (RF; i.e., the number of doublings relative to that of nontargeted control strains) of each strain during pooled growth with and without induction of the CRISPRi system using next-generation sequencing. This approach was reproducible (Fig. S2), and because it uses the sgRNA sequence as a barcode, it was not affected by cross-contamination.

10.1128/mBio.02561-21.2 FIG S2 Reproducibility of relative fitness measurements. Measurements for relative fitness were highly reproducible under both uninduced (A) and induced (B) conditions. Red dots represent control sgRNAs. Download FIG S2, PDF file, 1.1 MB. Copyright © 2021 Silvis et al. 2021 Silvis et al. https://creativecommons.org/licenses/by/4.0/ This content is distributed under the terms of the Creative Commons Attribution 4.0 International license.

Slight (basal CRISPRi, no IPTG) knockdown of a majority of essential genes (54%; 152/282 strains) resulted in a significant fitness defect (>3 standard deviations [SD] below the nontargeting controls) (Fig. 1C). In contrast, slight knockdown of nonessential genes seldom caused a significant fitness defect (7%; 13/187 strains) (Fig. 1C). Despite the large fraction of essential-gene knockdown strains with a significant fitness defect, the magnitude of the effect was small (median RF_ess_ = 0.98) (Fig. 1C). Only 53 essential gene knockdown strains exhibited a fitness defect ≥10% (RF < 0.9), indicating that many essential gene products are present in excess in wild-type cells, likely to buffer against environmental fluctuations, as previously suggested by pooled and arrayed approaches in E. coli, B. subtilis, and other species (8, 10, 15, 18, 20).

Strong (fully induced CRISPRi, 1 mM IPTG) knockdown of most essential genes caused significant fitness defects relative to the nontargeting controls (80%; 225/282 strains), and these defects were substantial in magnitude (median RF_ess_ = 0.73) (Fig. 1D). In contrast, knockdown of only a small fraction of nonessential genes (16%; 30/187 strains) caused reduced relative fitness even with induction (median RF_non-ess_ = 1.00) (Fig. 1D), consistent with the general lack of growth phenotypes in most viable E. coli deletion strains in rich media without inhibitory chemicals (1, 2, 21).

Recent studies identified sequence determinants of sgRNA toxicity (22) and efficacy (23). However, we found that neither sgRNA toxicity (Fig. S3A and B) nor sgRNA activity (Fig. S3C to F) was significantly correlated with relative fitness in either the induced or uninduced condition, suggesting that these effects did not significantly affect the observed phenotypes. Taken together, these data highlight the nuanced growth effects of CRISPRi repression of essential genes and confirm that our system is specific and well calibrated to study the effects of both slight and strong essential-gene knockdown.

10.1128/mBio.02561-21.3 FIG S3 Our CRISPRi strains are unaffected by the “bad-seed” effect, and sgRNA performance is not a driving factor in observed phenotypes. (A and B) There is no correlation between the bad-seed effect, quantified as the difference between the mean E18 (high *dcas9* strain) and mean E75 (moderate *dcas9* strain) fitness score of all sgRNAs with the same 5-nucleotide seed from Supplementary Data 3 in reference 22, and the relative fitness (RF) in our pooled screen without (A) or with (B) CRISPRi induction. (C and D) Predicted sgRNA activity (calculated using the model from reference 23) is not correlated with RF without (C) or with (D) CRISPRi induction. (E and F) Predicted sgRNA activity from is not correlated with the gene-normalized efficacy of our sgRNAs in pooled screens from references 13 (E) and 68 (F). Download FIG S3, PDF file, 1.4 MB. Copyright © 2021 Silvis et al. 2021 Silvis et al. https://creativecommons.org/licenses/by/4.0/ This content is distributed under the terms of the Creative Commons Attribution 4.0 International license.

### Growth consequences of slight knockdown of essential genes.

**FIG 2:**
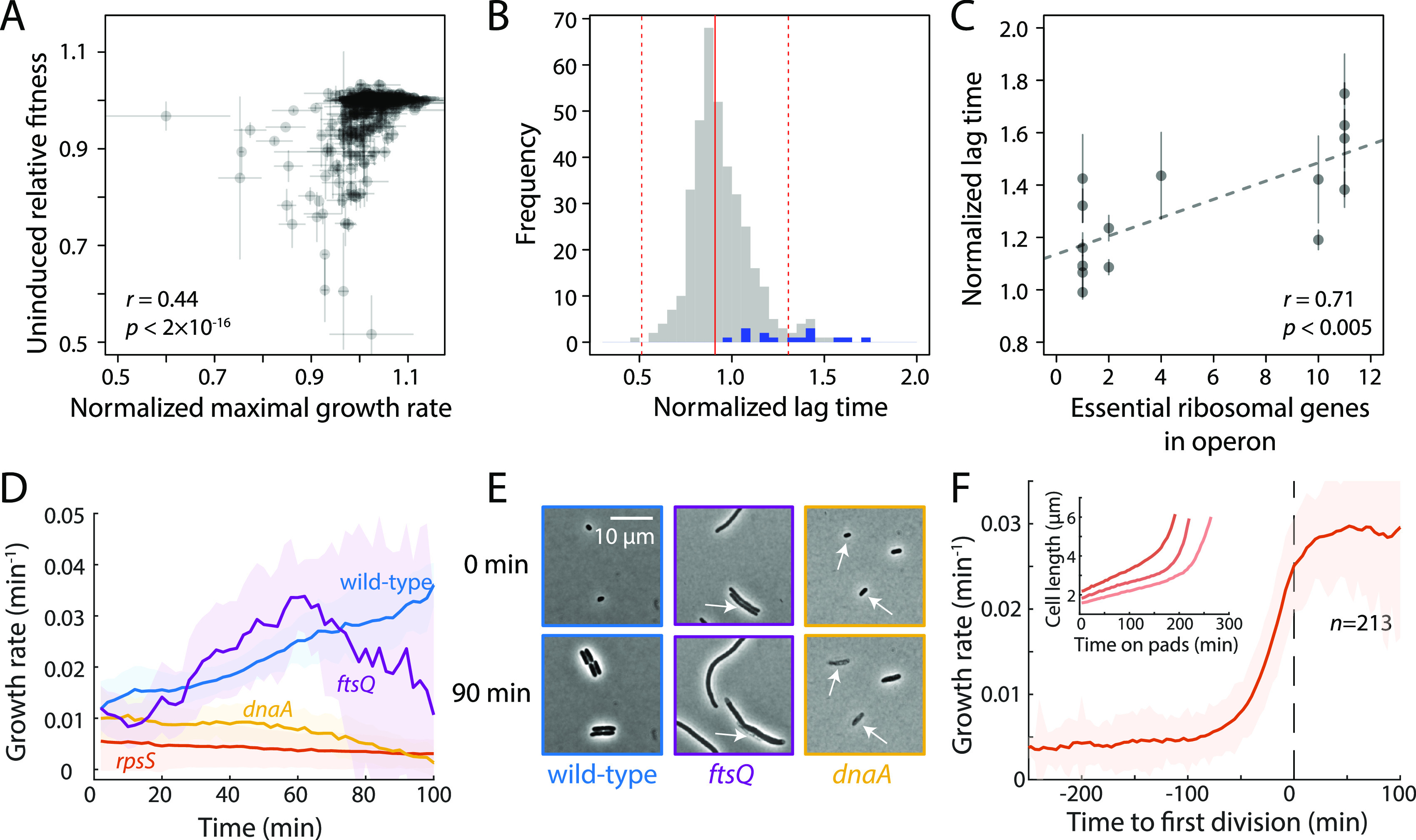
Strains with increased population-level lag time exhibit a range of single-cell phenotypes. (A) The maximum growth rate of strains grown individually without induction is correlated with their relative fitness in the pooled screen. Error bars represent the standard deviations (SD) for two biological replicates. (B) Histogram of lag times for CRISPRi strains normalized to WT. The solid red line indicates the median, and the dashed red lines indicate ±3 SD from the median, estimated from the median absolute distance. Blue bars denote ribosomal protein (*rps* and *rpl*) genes. (C) Normalized lag time is correlated with the number of essential ribosomal genes in the operon. The line denotes the linear best fit. (D) Single-cell instantaneous growth rates (1/*V dV*/*dt*) for three mutants with increased population-level lag times exhibited defects compared to wild-type after dilution of stationary-phase cells onto agarose pads with fresh medium. (E) The *ftsQ* and *dnaA* knockdown strains exhibited heterogeneity and growth defects during outgrowth from stationary phase. Most *ftsQ* knockdown cells first elongated, similar to the wild type, but later slowed growth (D) and failed to divide. A subset of *ftsQ* knockdown cells also lysed (white arrow). Many *dnaA* knockdown cells exhibited lysis (white arrows), and the remaining cells exhibited a lower growth rate than the wild-type (D), which increased only after 2 to 3 h (Fig. S4C). (F) *rpsS* cells increased growth rate concurrently with their first division after stationary-phase outgrowth. (Inset) Each *rpsS* cell first grew slowly and linearly for >100 min before transitioning into exponential-like growth. Data for three representative cells are shown. Data in panels D and F are means and SD.

Our pooled competitive growth assays identified a number of strains with significant fitness defects during growth with basal CRISPRi induction. However, these endpoint assays lacked the temporal resolution to determine whether decreased fitness is due to a lower maximal growth rate or a longer lag phase (see Materials and Methods). To determine the growth dynamics of our E. coli essential-gene knockdown strains with basal CRISPRi knockdown, we grew each of our strains overnight in LB, then diluted them into fresh medium, and measured their optical density (OD) in a plate reader as they resumed growth (Materials and Methods). Results were reproducible (Fig. S4A and B), and normalized maximal growth rates of individual strains were correlated with their fitness (RF) in the pooled screen (*r = *0.44; *P < *2 × 10^−16^) (Fig. 2A; Table S2), suggesting that most RF defects were due to slower growth rather than a longer lag phase.

10.1128/mBio.02561-21.4 FIG S4 Reproducibility of growth measurements. (A and B) Population-level measurements for maximum growth rate (A) and lag time (B) were reproducible. (C) The growth rate of the *dnaA* knockdown strain accelerated after 2 to 3 h, with large variability across cells. Download FIG S4, PDF file, 0.9 MB. Copyright © 2021 Silvis et al. 2021 Silvis et al. https://creativecommons.org/licenses/by/4.0/ This content is distributed under the terms of the Creative Commons Attribution 4.0 International license.

10.1128/mBio.02561-21.7 TABLE S2 Pooled and individual growth rates of E. coli CRISPRi library with and without induction of the CRISPRi system. Download Table S2, XLSX file, 0.2 MB. Copyright © 2021 Silvis et al. 2021 Silvis et al. https://creativecommons.org/licenses/by/4.0/ This content is distributed under the terms of the Creative Commons Attribution 4.0 International license.

Only 16 E. coli essential-gene knockdown strains exhibited significantly longer lag time (>3 SD higher than the median) (Fig. 2B; Table S2), a majority of which targeted ribosomal proteins (8/16 strains) and other translation-related genes (2/16 strains). The remainder targeted genes involved in cell division and DNA synthesis (*dnaA*, *ftsQ*, and *tmk*), and lipoprotein processing (*lolCD* and *lgt*). A previous study of the E. coli Keio knockout collection grown on agar plates identified long-lag phenotypes in strains with deletions of tRNA modification (e.g., *tusABCDE* and *rnt*) and ribosome maturation (e.g., *rimM*, *rbfA*, and *rgsA*) genes and hypothesized that a general decrease in translation efficiency may be responsible for this phenotype (24). Our observation that a majority (8/16 strains) of assayed essential ribosomal gene knockdown strains (*rps*, and *rpl*) exhibited long lag time strongly supports this hypothesis. Furthermore, when all essential ribosomal genes in our library were considered, lag time was correlated with the number of essential ribosomal genes in the targeted operon (*r *= 0.71; *P < *0.005) (Fig. 2C). Strains targeting the largest ribosomal protein operons (the S10 and L14 operons) exhibited the longest lag times, potentially due to polar effects of CRISPRi knockdown (25) affecting the expression of multiple ribosomal proteins and thus amplifying the effect of these knockdowns on translational capacity.

To understand the manifestation of long lag times at the single-cell level, we selected three knockdown strains with long lag times, the *rpsS*, *dnaA*, and *ftsQ* strains, and imaged their emergence from stationary phase on agar pads while quantifying the instantaneous growth rates of individual cells. The instantaneous growth rate of individual cells was calculated as 1/*V dV/dt*, which describes the relative rate of volume expansion, and is consistent with bulk measurements such as OD and CFU for steady-state cultures (26). During imaging, wild-type cells gradually increased their growth rate as they emerged from stationary phase until they reached ∼0.03 min^−1^ (∼23 min doubling time), usually after ∼1.5 h (Fig. 2D). *ftsQ* knockdown cells similarly increased their growth rate, but for only ∼60 min, after which growth rate plateaued and decreased (Fig. 2D). Decreased growth rate was associated with filamentation and some lysis (Fig. 2E). In contrast, many *dnaA* knockdown cells lysed by 1.5 h (Fig. 2E), and the cells that resumed growth exhibited low growth rates until ∼2 h postdilution and reached maximum growth rates only after >3 h (Fig. S4C).

The *rpsS* knockdown strain exhibited a previously uncharacterized behavior as it emerged from stationary phase: cells grew for several hours with slow, approximately linear kinetics (decreasing instantaneous growth rate with time) followed by a transition to exponential growth (Fig. 2F, inset). Interestingly, the transition to rapid growth tended to occur in a fixed window ∼30 min prior to cell division (Fig. 2F), suggesting that cell division proceeds only after a reversal of slowdown. This long-lag phenotype persisted upon repeated regrowth and dilution, indicating that depletion of ribosomal proteins in stationary phase rather than genetic suppression of CRISPRi activity likely underlies this phenomenon. These results highlight the variability of single-cell phenotypes that can underlie slow growth caused by slight knockdown of essential genes and demonstrate a requirement for translational capacity during the transition from stationary to exponential growth. It is likely that a comprehensive analysis of single-cell growth rates and morphologies will identify both additional genes and phenotypes.

### Morphological profiling identifies genes required for normal cell shape.

**FIG 3:**
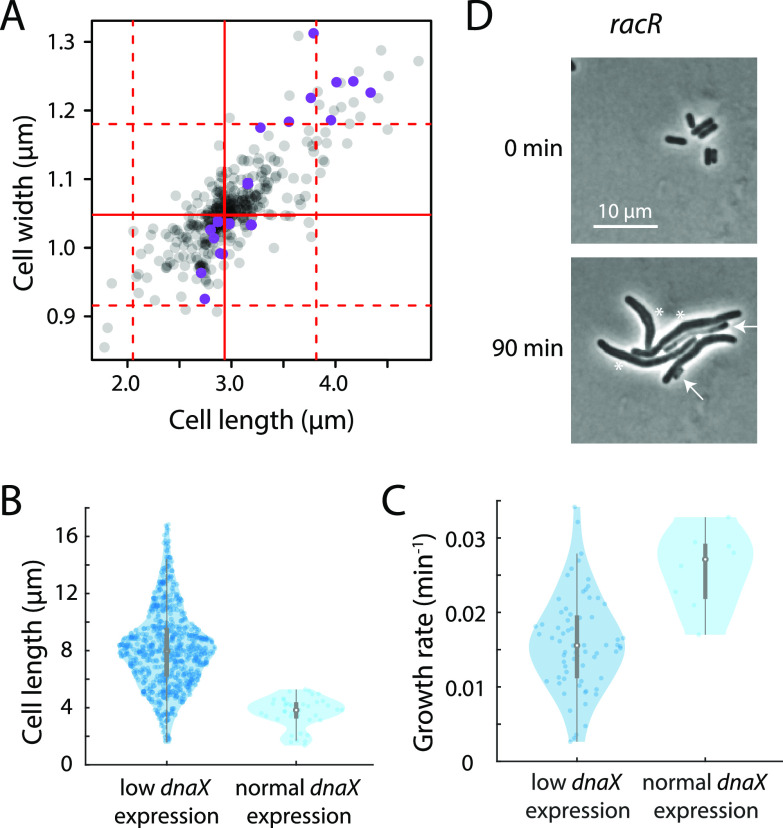
Heterogeneity in cell length can be linked to expression level or growth defects. (A) Cell length and width corrected for plate effects (Materials and Methods) of all strains in the uninduced condition. Solid red lines indicate the median width and length, and the dashed red lines indicate ±3 standard deviations (SD) from the median, estimated from the median absolute distance. Purple dots are outer membrane-related genes. (B and C) During outgrowth from stationary phase, *dnaX* cells exhibited heterogeneity in *dnaX* expression. Cells with low *dnaX* expression filamented (B) and grew more slowly (C) than cells with higher *dnaX* expression. (D) The *racR* strain exhibited filamentation, bulging (white asterisks), and lysis (white arrows) during outgrowth.

Decreased growth rate is just one reflection of altered cellular homeostasis caused by the knockdown of essential genes. To more broadly determine how partial knockdown affects cellular physiology, we quantified the cellular dimensions of all strains in our library in the presence of basal CRISPRi knockdown after 3.5 h of growth in fresh LB. After correcting for plate effects (see Materials and Methods), most strains exhibited wild-type length and width (90%; 310/346 strains) (Fig. 3A; Table S3). A total of 23 strains were significantly wider than the median (>3 SD wider than the median) and 25 strains were significantly longer (Table S3). A majority of strains with altered dimensions (55%; 17/31 strains) were both significantly longer and significantly wider (Fig. 3A), suggesting a general breakdown of morphological homeostasis in these strains.

10.1128/mBio.02561-21.8 TABLE S3 Shape data for E. coli CRISPRi library measured with and without induction of the CRISPRi system. Download Table S3, XLSX file, 0.06 MB. Copyright © 2021 Silvis et al. 2021 Silvis et al. https://creativecommons.org/licenses/by/4.0/ This content is distributed under the terms of the Creative Commons Attribution 4.0 International license.

As reported in previous studies (21), we found that length heterogeneity within a strain (quantified by the coefficient of variation [CV]) was much greater than width variability (Table S3). To better understand sources of length variability, we performed time-lapse imaging of two strains with high length variability in which a monocistronic operon was targeted (*racR* and *dnaX*). Imaging of the *dnaX* (DNA polymerase III τ subunit) knockdown strain revealed bimodality in both growth rate and cell length. This heterogeneity likely reflects noise in *dnaX* knockdown levels, as imaging of *dnaX* cells containing a green fluorescent protein (GFP) reporter of *dnaX* expression (27) revealed that low-expression cells were longer (Fig. 3B) and grew more slowly than high-expression cells (Fig. 3C). The *racR* strain, depleted of a repressor of nearby toxin genes (28), exhibited slow single-cell growth, as well as bulging, filamentation, and some explosive lysis (Fig. 3D). Length variability likely reflected differences in the timing of toxin induction. These findings suggest that a variety of single-cell phenotypes can be responsible for the variability in length caused by slight knockdown of essential genes.

The knockdown strain targeting the known modulator of cell width *mreB* (29) exhibited significant increases in width, as did strains targeting genes implicated in the maintenance of outer membrane integrity, such as *lolCDE*, *lspA*, *lpxB*, *kdsB*, and *lgt* (Fig. 3A). Indeed, knockdown strains targeting outer membrane function genes were significantly enriched among wide strains (*P < *5 × 10^−5^, hypergeometric test). A previous study quantified the morphology of all nonessential gene deletions (21) and found that the deletion of genes involved in the synthesis of the enterobacterial common antigen (ECA) caused significant increases in cell width, potentially by sequestering the undecaprenyl phosphate lipid carrier also required by peptidoglycan synthesis (30, 31). Our finding that slight depletion of genes involved in lipoprotein trafficking (*lolCDE*) and modification (*lgt*), which do not involve an undecaprenyl phosphate lipid carrier, also cause a significant increase in cell width suggests that a different mechanism may be involved, perhaps related to the mechanical properties of the outer membrane (32) or to the depletion of lipoproteins involved in cell wall synthesis. In addition to genes with direct roles in shape determination, we also identified genes likely to have indirect effects on cell shape, including *ribA*, *pth*, *holB*, *panC*, and *ileS* (Table S3). Although operon effects are likely responsible for some of these phenotypes (e.g., *holB* is upstream of *mltG*, *ileS* is upstream of *lspA*), others are likely due to biologically meaningful indirect effects.

### Terminal morphologies reveal novel modulators of stress pathways.

**FIG 4:**
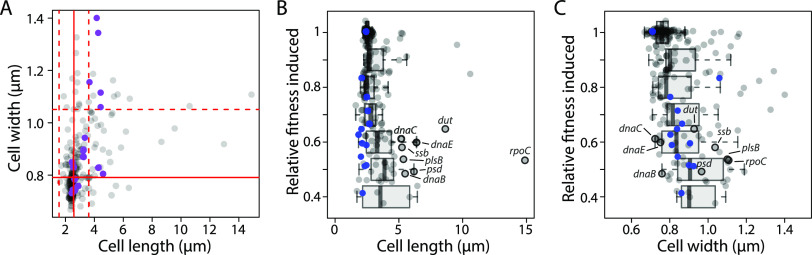
Fully induced CRISPRi targeting of essential genes results in morphological phenotypes characteristic of stress responses. (A) Median length and width for all strains after full induction of CRISPRi. Purple dots represent outer membrane-related genes. Solid red lines indicate the median width and length, and the red dashed lines indicate ±3 SD from the median, estimated from the median absolute distance. (B and C) Altered cell length and width were associated with reduced relative fitness, suggesting a connection between morphological defects and growth. Box plots show the distributions of median strain length and width within bins of 0.1 RF. tRNA synthases, represented by blue dots, exhibited short length (B) but normal width (C), consistent with the induction of the stringent response. Genes discussed in the text as exhibiting low relative fitness and filamentation are outlined by black circles.

Measurements of cellular morphology at a low level of CRISPRi activity (basal induction) revealed direct modulators of cell size. To determine how strong (fully induced) depletion of essential genes affects cellular physiology, we quantified the morphology of each strain after 5.5 h of growth in fresh LB with 1 mM IPTG. As expected, more strains exhibited strong cell dimension phenotypes (26%; 59/239) than in the absence of inducer (Fig. 4A). A total of 26 strains were significantly wider than the median (>3 SD wider than the median), and 47 strains were significantly longer (Table S3). Atypical dimensions were associated with decreased fitness (RF) in the pooled screen (Fig. 4B and C) and therefore may represent the indirect effects of stress responses pathways, such as the SOS response and the stringent response, which are activated by potentially lethal challenges such as strong depletion of essential genes (33, 34).

Filamentation due to cell division inhibition is canonically associated with the SOS response, a deeply conserved pathway that senses DNA damage and halts cell division until the damage can be repaired (35). Of the 29 strains that grew poorly (RF_induced_ < 0.7) and were significantly longer (>3 SD more than the median; Table S3), we focused on the 10 with the clearest filamentation phenotypes (median length > 5 μm). Consistent with SOS induction causing filamentation, depletion of genes directly or indirectly involved in DNA replication, such as *dnaBCE*, *dut*, and *ssb*, was associated with strong filamentation phenotypes. However, not all strong filamentation resulted from SOS induction. For example, knockdown of *rpoC* caused the strongest filamentation phenotype in our screen (median length > 14 μm) (Table S3). Inhibition of RpoC using a cyclopeptide inhibitor (36) was previously shown to cause SOS-independent filamentation (37). Depletion of the phospholipid synthesis genes *psd* and *plsB* also caused filamentation, consistent with previous studies (38), although whether these phenotypes manifest through the SOS response remains to be determined.

Strains also exhibited phenotypes consistent with activation of the stringent response. The stringent response is induced by uncharged tRNAs and prepares cells for starvation by downregulating translation, upregulating amino acid synthesis, and arresting the cell cycle (39). Activation of the stringent response is associated with decreased cell length (40–42). We identified 16 strains (Fig. 4B) with substantial growth rate decreases (RF_induced_ < 0.7) and decreased length (below the median of all strains) (Fig. 4B). Consistent with these phenotypes resulting from stringent response activation, 8 of these 16 strains target tRNA synthases and 2 target amino acid synthesis genes (*dapD* and *glyA*). In addition to these strains, strains targeting *adk*, *ribA*, *ribC*, *kdsA*, *yihA*, and *ppa* exhibited a similar phenotype. *adk*, which encodes adenosine kinase, was recently implicated as a potential activator of the stringent response (43). Depletion of genes involved in riboflavin synthesis (*ribA* and *ribC*) likely activates the stringent response through one or more of the 38 E. coli flavoenzymes, many of which are metabolic enzymes (44). No studies currently implicate genes involved in lipopolysaccharide (LPS) synthesis (*kdsA*), inorganic pyrophosphatase (*ppa*), or the cell cycle-related GTPase (*yihA*) as playing a role in the stringent response. It remains to be determined if the small-cell-size phenotypes observed for these strains are due to indirect activation of the stringent response or to other stress response pathways.

### *mreB* is subject to negative transcriptional feedback.

**FIG 5:**
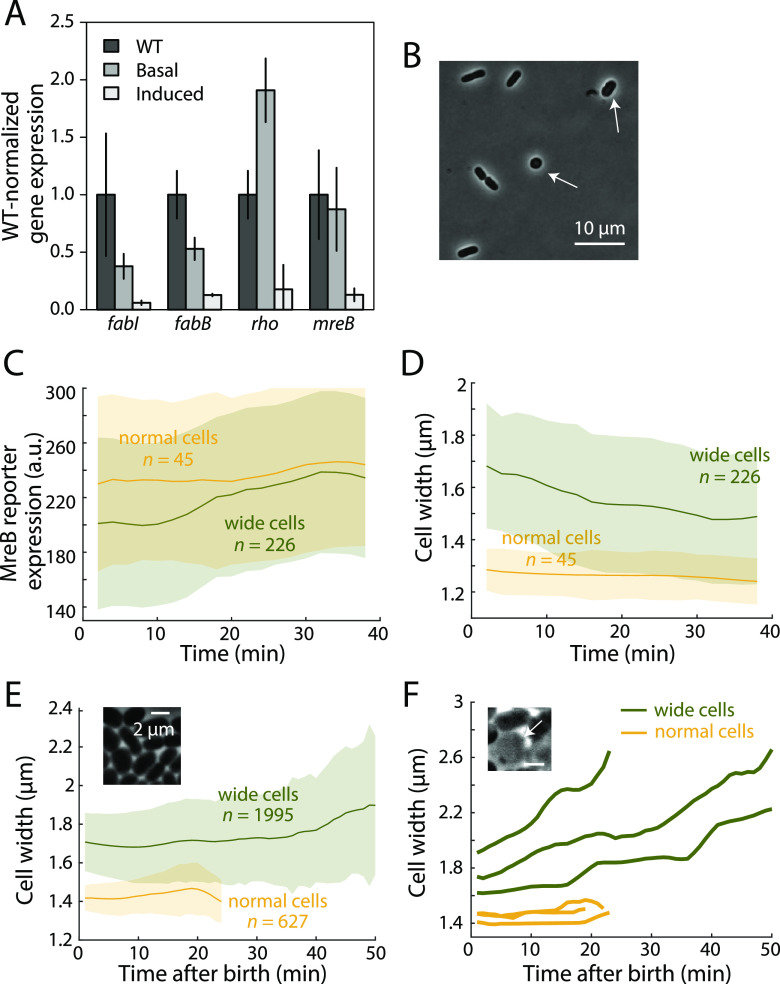
Transcriptional feedback on *mreB* expression is critical for width control and cell viability. (A) RT-qPCR measurements of the effect of basal and full CRISPRi induction on *fabI*, *fabB*, *rho*, and *mreB* expression normalized to the expression of those genes in the wild-type strain. Expression levels of *rho* and *mreB* were not inhibited by basal induction of CRISPRi but were decreased by the fully induced system. (B) Slight knockdown (no IPTG) of *mreB* led to shape heterogeneity in which some cells were substantially wider (white arrows). (C and D) Time-lapse imaging of the *mreB* knockdown strain with a TSS1 reporter. Wider cells exhibited lower MreB expression, but MreB expression increased in these cells during the imaging (C), concurrent with a decrease in cell width (D), demonstrating transcriptional-level feedback. (E) In a strain with *mreB* under the control of an inducible IPTG-regulated synthetic promoter (to eliminate transcriptional feedback), wide cells continued to increase in width when cultured in a microfluidic device with continuous flow of fresh nutrient. (Inset) Cells in the microfluidic device exhibited large variations in cell width. Data in panels C to E are means and SD. (F) Typical cell width traces for the experiment shown in panel E. While cells with normal width grew and divided normally, once cell width increased to >1.6 μm, they continued to increase in width, failed to divide, and eventually lysed. (Inset) A cell that lysed due to failure in width control.

Unlike other approaches for modulating essential gene expression, such as promoter replacement (45), CRISPRi modulation of gene expression is responsive to native transcriptional feedback circuits (14, 46). We focused our attention on the actin homolog *mreB*, which has no characterized transcriptional feedback (47, 48) but exhibits strikingly different susceptibility to knockdown in B. subtilis and E. coli (15). Whereas *mreB* knockdown in B. subtilis results in substantial lysis after 10 generations, similar *mreB* knockdown in E. coli does not substantially affect fitness (15). To determine if negative transcriptional feedback is responsible for the robustness of E. coli to *mreB* knockdown, we assayed the expression of *mreB*, as well as genes with (*rho*) (49) and without (*fabB/fabI*) known homeostatic feedback regulation in the presence of various degrees of CRISPRi knockdown using reverse transcription-quantitative PCR (RT-qPCR) (see Materials and Methods). As expected, both *fabB* and *fabI* exhibited monotonically decreasing knockdown as the CRISPRi system was induced (Fig. 5A). In contrast, *rho* and *mreB* expression levels were not affected by slight (uninduced) CRISPRi knockdown. Full induction of the CRISPRi system resulted in substantial downregulation of *rho* and *mreB* (Fig. 5A), suggesting that the sgRNAs targeting *rho* and *mreB* are functional, and that the lack of observed knockdown in the uninduced experiment is due to the activation of native feedback circuits, which can be overcome with sufficiently high levels of repression.

To determine if *mreB* is subject to transcriptional feedback, we constructed a fluorescent reporter in which the *mreB* promoter drives *gfp* expression from an upstream region containing the native promoter and the 5′ end of the gene (27) including the CRISPRi-targeted spacer (Fig. S5A) and performed time-lapse imaging of log-phase cells harboring this reporter (without CRISPRi induction). Slight *mreB* knockdown led to heterogeneous cell widths (Fig. 5B). We grouped cells by their width at time zero into two groups: those with wild type-like widths (cell width < 1.4 μm) and cells much wider than the wild type (cell width ≥ 1.4 μm). Wide cells exhibited lower *mreB* expression (Fig. 5C), which gradually increased during imaging, concurrent with a decrease in width (Fig. 5C and D). Because the *mreB* promoter contains three putative transcriptional start sites (TSSs) (50), we constructed two additional reporters, encompassing truncated versions of the promoter. All three fluorescent reporters behaved similarly (Fig. 5C; Fig. S5B and C). These data are consistent with a transcriptional feedback circuit regulating MreB expression and suggest that the first TSS is sufficient for feedback regulation.

10.1128/mBio.02561-21.5 FIG S5 Transcriptional feedback of *mreB* is essential for cell width maintenance. (A) Schematic depicting the design of fluorescence reporters for *mreB* expression (knockdown reporter) and feedback (feedback reporter). (B and C) Time-lapse imaging of the *mreB* knockdown strain with TSS12 (B) and TSS123 (C) reporters showed feedback dynamics similar to that of the TSS1 reporter (Fig. 5C). (D) In the *mreB* knockdown strain with a feedback reporter, wider cells consistently had higher levels of feedback than cells with normal width. (E and F) When treated with A22 or amdinocillin, cell width increased (E) along with MreB expression (F), indicating the activation of homeostatic feedback circuits. (G) A strain with *mreB* under the control of an IPTG-inducible promoter can undergo stable passage at different IPTG levels, and cell width was dependent on the levels of induction. The width of the IPTG-inducible strain was between the widths of the wild type and the *mreB* CRISPRi strain. (H) The IPTG-inducible *mreB* strain failed to maintain width during continuous exponential growth in a microfluidic device at 10 μM IPTG, similar to its behavior at 100 μM IPTG (Fig. 5E). Data in panels B to H are means and 1 SD. Download FIG S5, PDF file, 0.5 MB. Copyright © 2021 Silvis et al. 2021 Silvis et al. https://creativecommons.org/licenses/by/4.0/ This content is distributed under the terms of the Creative Commons Attribution 4.0 International license.

To confirm that the observed increase in fluorescence was due to feedback, we constructed and measured a feedback reporter, which was identical to our full-length transcriptional reporter except that the protospacer-adjacent motif (PAM) targeted by the CRISPRi system was mutated (Fig. S5A). This reporter is therefore not affected by CRISPRi, and hence, its expression reports only upregulation in response to perturbation of expression at the native locus. Wide cells exhibited higher expression of this reporter (Fig. S5D), confirming that *mreB* expression was responsive to cell width.

Finally, to determine if the putative feedback mechanism senses the level of *mreB* expression or the activity of the elongation system, we measured the expression of the full-length *mreB* reporter in the presence of A22 or amdinocillin. A22 causes cell widening by depolymerizing MreB filaments and inhibiting MreB activity but does not directly alter *mreB* expression (51). Amdinocillin targets PBP2, a component of the essential cell wall synthase of the elongation machinery, which also leads to cell widening via its interactions with the cell wall synthesis machinery and MreB (51). Both A22 and amdinocillin treatment resulted in increased expression of the full-length *mreB* reporter concurrent with cell widening (Fig. S5E and F), suggesting that transcriptional regulation of the *mreB* promoter occurs in response to elongation system activity rather than *mreB* expression. The mechanistic details of this newly discovered response remain to be elucidated.

### Transcriptional feedback is critical for cell shape maintenance and stability.

We next sought to determine the importance of transcriptional feedback on *mreB* for cell width maintenance. To do so, we eliminated normal transcriptional controls by using a strain in which *mreB* (but not its promoter, which drives *mreCD* expression) is deleted from the chromosomal locus and provided on a plasmid under the control of an inducible IPTG-regulated synthetic promoter (52). In the presence of 10 μM or 100 μM IPTG, this strain did not exhibit growth defects during normal daily passaging. The width of this strain depended on external IPTG levels and was between that of the wild-type strain and that of the uninduced *mreB* CRISPRi strain (Fig. S5G), suggesting that IPTG-induced MreB expression was somewhat less than in the wild-type strain but more than in the *mreB* CRISPRi strain. However, when we attempted to propagate this strain in steady-state exponential growth in a microfluidic device with continuous flow of fresh medium and 100 μM IPTG, cells exhibited variations in cell width after several doublings (Fig. 5E, inset). Once these cells increased in width beyond ∼1.6 μm, they continued to widen, failed to divide, and eventually lysed (Fig. 5E and F). Similar dynamics were observed at lower IPTG concentrations, where cells lost width control more rapidly (Fig. S5H). In contrast, *mreB* CRISPRi knockdown cells wider than 1.6 μm were able to counteract continued widening and avoid lysis (Fig. 5D). Cell lysis and loss of width control were also observed in test tubes when the IPTG-inducible *mreB* strain underwent repeated dilutions to maintain an OD of <0.1, indicating that the observed phenotype is not specific to growth in microfluidic devices. These data suggest that transcriptional feedback at the native *mreBCD* locus is necessary for the stable maintenance of cell width and that disrupting this regulation leads to fluctuations that disrupt E. coli cell width, especially during extended periods of steady-state, exponential growth.

## DISCUSSION

Here, we constructed an arrayed library of chromosomally encoded CRISPRi strains targeting the essential genes of the model bacterium E. coli and used it to study their functional importance. Our CRISPRi system, which was designed with a constitutively expressed *dcas9* and an inducible sgRNA for fast turn-on, is flexible and allows the use of different *dcas9* constructs. Although numerous CRISPRi studies have been performed in E. coli and other Gram-negative bacteria, to date all arrayed CRISPRi libraries have been constructed in Gram-positive species (12). Because many differences between Gram-positive and Gram-negative bacteria are found in essential structures such as the outer membrane and the divisome, our library will be a powerful resource for the microbiology community.

Previous work suggested that decreases in translational capacity could result in long lag times, but the essentiality of most ribosomal proteins meant that this hypothesis could not easily be tested (24). We showed that slight knockdown of essential ribosomal proteins is sufficient to substantially lengthen lag phase (Fig. 2), emphasizing the role of translational capacity during growth transitions and explaining a previous observation that ribosomal protein genes are upregulated very early in lag phase (53). Reduced translational capacity during lag phase led to a surprising phenotype: for several hours, cells grew with linear rather than exponential dynamics. This behavior was followed by a transition to exponential growth, which generally occurred ∼30 min before cell division (Fig. 2F). Determining how translational capacity is linked to cell division during growth transitions will open exciting new windows into regulation of the bacterial cell cycle.

Profiling the cellular dimensions of our knockdown strains under slight (basal) and strong (fully induced) knockdown revealed previously unrecognized aspects of bacterial physiology. Under slight knockdown, most strains were able to grow at near-wild-type rates. As a result, we identified strains in which cell shape was directly impacted by knockdown, such as those targeting lipoprotein export genes (*lolCDE* and *lgt*), which exhibited high cell width potentially caused by changes to the mechanical properties of the outer membrane or by decreases in the levels of lipoproteins involved in cell wall synthesis. Fully inducing the CRISPRi system led to more extreme morphological defects, which were associated with decreased growth rates and presumably represented the action of cellular stress responses. For example, strong knockdown of genes involved in DNA synthesis (*dnaBCE*, *dut*, and *ssb*) led to filamentation, likely by activating the SOS response. Similarly, knockdown of many tRNA synthases led to short cells, likely due to activation of the stringent response. Further studies to determine which stress responses are induced in response to the depletion of specific essential processes will deepen our understanding of the myriad ways bacteria survive antibiotic and chemical challenges.

Previously, we found that E. coli could tolerate significant knockdown of the essential operon *mreBCD*, while B. subtilis could not (15). Our E. coli results were supported by a study that found limited knockdown when targeting *mreC* using a different CRISPRi system (54) and by the limited fitness effects of CRISPRi repression of the Enterobacter cloacae *mreCD* genes (55). We speculated that the robustness of the *mreBCD* operon to CRISPRi may be due to transcriptional feedback (15), which can mitigate or even overcome CRISPRi repression (14, 46). Using a series of fluorescent feedback and expression reporters, we found that transcriptional feedback was responsible for mitigating CRISPRi repression of *mreB*. Moreover, analysis of a strain in which *mreB* is placed under the control of a synthetic inducible promoter suggested that feedback control of the *mreBCD* locus is required for the stability of width control. In E. coli, the *mreBCD* operon is negatively regulated by BolA (47), which is conserved in Gram-negative but not Gram-positive bacteria (56). However, BolA appears unlikely to be responsible for the observed transcriptional feedback, because its deletion is viable and does not alter cell shape in exponential phase (21). Thus, our data suggest that a hitherto-undiscovered and critical regulatory mechanism is responsible for maintaining consistent MreB activity in the face of external perturbations.

Arrayed libraries of CRISPRi strains targeting essential genes have already demonstrated their usefulness in Gram-positive bacteria, greatly contributing to our understanding of essential gene function and the interplay between essential processes (8–11). We anticipate that the E. coli library described here will set the stage for similarly powerful advances in Gram-negative bacteria.

## MATERIALS AND METHODS

### Microbes.

Escherichia coli strains were cultured in LB medium at 37°C. Antibiotics used were gentamicin (10 μg/ml), chloramphenicol (25 μg/ml), kanamycin (30 μg/ml), A22 (10 μg/ml), and amdinocillin (1 μg/ml).

### CRISPRi library design.

sgRNAs were designed to target genes in E. coli BW25113 for which there is some evidence of essentiality in published data sets, as summarized in Table S1. sgRNAs were designed to target within each gene’s ORF near the 5′ end and bind the nontemplate strand, and sgRNAs with multiple potential binding sites were avoided, as previously described (8). sgRNA design scripts are publicly available at https://github.com/traeki/sgrna_design.

### CRISPRi strain construction.

The lambda att-integrating plasmid pCAH63 (19) was modified to contain an sgRNA expression cassette to generate pCs-550r in the following steps: the sgRNA constant region was cloned from pgRNA-bacteria (Addgene number 44251) (7); the terminators L3S3P22 and L3S2P21 were cloned up- and downstream, respectively, to flank the sgRNA cassette; and the sgRNA promoter was changed from BBa_J23119 to PlLac-O1 (57). New 20-nucleotide spacers were cloned into pCs-550r via inverse PCR (58), Sanger sequenced, and transformed into E. coli BW25113 harboring pINT-ts to promote integration at lambda att (19) using CaCl_2_ competence and selecting for chloramphenicol resistance.

High-efficiency conjugation was used to transfer *dcas9* from the chromosome of a donor strain to the chromosome of a sgRNA-encoding recipient strain. A pseudo-Hfr strain isogenic to BW25113 carries the transfer region from F and a spectinomycin marker integrated downstream of *rhaM* (59). The *dcas9* donor strain was constructed by integrating *dcas9* and a gentamicin resistance marker at the Tn*7* att site (60), adjacent to the origin of transfer, using the Mobile-CRISPRi triparental mating strategy (55). To clone the Tn*7* cassette plasmid, *dcas9* was amplified from pdCas9-bacteria (Addgene number 44249) under the control of the synthetic promoter BBa_J23105 (http://parts.igem.org/). Conjugation was performed on LB plates by mixing the *dcas9* donor and sgRNA recipient in equal ratios, incubating for 5 h at 37°C, pinning onto double-selection plates (chloramphenicol plus gentamicin), and growing overnight. Single colonies from each conjugation mix were isolated by streaking onto double-selection plates.

### RFP strain construction.

The *rfp* cassette including a Kan^r^ marker was PCR amplified from the entry vector used to construct the previously described red fluorescent protein (RFP) reporter strain (plasmid, pSLQ1232; strain, MG1655 *nfsA*::PlLac-O1-*mrfp*) (7). The *rfp* promoter was changed from PLlac-O1 to a minimal synthetic promoter (BBa_J23119; http://parts.igem.org/Main_Page) to create pSLQ1232-P541-rfp and then integrated into BW25113 at *nfsA* by λ*red* recombineering (61) and selecting for kanamycin resistance. Promoter variants were cloned along with *dcas9* into the Tn*7* cassette plasmid, and triparental mating was used to introduce *dcas9* cassette into the chromosome at Tn*7* att, as described above.

### Flow cytometry to quantify knockdown and reporter activities.

Flow cytometry analysis of the RFP reporter strains was performed as described in reference 62 with minor modifications; strains were initially inoculated from single colonies and grown for ∼5 h before dilution instead of overnight. Data were collected on an LSRII flow cytometer (BD Biosciences) using the yellow/green laser (561 nm) and the phycoerythrin (PE)-Texas Red detector (610/20 nm). Data for at least 20,000 cells were collected, and median fluorescence values were extracted using FlowJo (FlowJo, LLC). Data from representative samples were plotted as histograms using FlowJo.

### CRISPRi library construction.

sgRNA plasmids were cloned, verified, and integrated into E. coli BW25113 as described for individual strains above. One isolate of each sgRNA recipient was stored by inoculating into 250 μl LB with chloramphenicol in deep 96-well plates, grown for 6.5 h, mixed with glycerol, and stored at −80°C. Arrayed sgRNA recipient libraries and the arrayed *dcas9* donor strain were pinned from glycerol stocks to separate LB agar plates using a ROTOR robot (Singer Instruments) and grown overnight. The arrayed recipient library was then mixed with the arrayed donor strain by pinning onto a new LB agar plate and then grown for 8 h to allow conjugation. Patches were mixed and transferred to a double-selection agar plate (gentamicin and chloramphenicol) using the ROTOR robot and grown overnight. Patches were each individually struck out on double-selection plates for single-colony isolation. To store the CRISPRi library, 2 isolates of each strain were inoculated in 250 μl LB with chloramphenicol and gentamicin supplemented with 0.2% glucose in deep 96-well plates, grown for 6.5 h, mixed with glycerol, and stored at −80°C in 96-well plates.

### CRISPRi library sequencing.

The sgRNA regions were first amplified from frozen stocks using round 1 forward and reverse primers (Table S1) and then cleaned up using an Exo-CIP PCR cleanup kit (NEB). The PCR products were diluted 1:50 and amplified again using custom primers containing Nextera adapters and indices. Samples were then pooled and cleaned using a NucleoSpin PCR cleanup kit (TaKaRa). Sequencing was performed on an Illumina NextSeq 500 system. Spacer sequences were extracted from fastq files and counted by exact matching to the designed library sequences.

### Pooled library construction.

To enable the use of deep sequencing to quantify relative fitness, an additional ∼50 nontargeting sgRNA plasmids were cloned and integrated into BW25113, as described above. Control sgRNA spacers were selected as a random subset from previously characterized control sgRNAs (15). Construction of the pooled library (all library sgRNAs plus control sgRNAs) was identical to that of the arrayed library, except that after the second double selection of the arrayed library, all patches were scraped from the agar plate, thoroughly mixed, and stored as glycerol stocks at −80°C.

### Pooled growth experiment.

To quantify the relative fitness of each CRISPRi strain, we enumerated the relative proportion of each sgRNA spacer in the mixed population by deep sequencing, before and after 15 doublings in saturating IPTG. Briefly, a single glycerol stock of the pooled library was fully thawed, inoculated into 10 ml LB at an OD_600_ of 0.01, and grown for 2.5 h (final OD_600_ ∼0.3). This culture was collected (10 ml, time [*t*] = 0) and used to inoculate replicate 4-ml LB cultures (with or without 1 mM IPTG) at an OD_600_ of 0.01, which were then repeatedly grown for 130 min to an OD_600_ of 0.3 (∼5 doublings) and back-diluted to 0.01 a total of 3 times (to allow 15 doublings). After growth back to an OD_600_ of 0.3, cultures were collected (4 ml) by pelleting (9,000 × *g* for 2 min) and stored at −80°C. The following day, genomic DNA was extracted using the DNeasy blood and tissue kit (Qiagen number 69506) with the recommended Gram-negative pretreatment and RNase A treatment. sgRNA spacer sequences were amplified from gDNA using Q5 polymerase (New England Biolabs number M0493S) for 14 cycles using custom primers containing TruSeq adapters and indices, followed by gel purification from 8% Tris-borate-EDTA (TBE) gels.

Spacer sequences were extracted from fastq files, counted by exact matching to expected library spacers, and their counts were normalized within each sample to control for read depth. We calculated the fitness as relative fitness (15), wherein the log_2_ fold change was normalized by the median log_2_ fold change of the control sgRNAs and adjusted by the number of doublings. All RF values are reported in Table S2.

### Population-level growth analyses.

To measure growth dynamics, overnight cultures were inoculated into 80 μl of fresh medium in a clear 384-well plate. The plate was covered with an optical film, with small holes poked at the side of each well to allow aeration. Incubation and OD measurements were performed with an Epoch 2 plate reader (BioTek) at 37°C with continuous shaking, and OD_600_ was measured at 8.5-min intervals. The instantaneous growth rates were calculated as the slope of ln(OD) with respect to time after smoothing using a moving average filter with a window size of 5. Maximum growth rate was calculated as the largest instantaneous growth rate. Lag time was defined as the duration before cells reaching half of their maximum growth rate.

To normalize per-strain growth rates and lag times due to inoculation effects, a 384-well plate containing only wild-type cells was grown and analyzed as described above. The maximum growth rate and lag time of each CRISPRi strain was compared to the median for the 10 wild-type replicates with the closest starting optical density.

### Morphological analyses.

Overnight cultures were inoculated into fresh medium and grown to log phase in multiwell plates. For microscopy images with a single time point, the MATLAB (MathWorks, Natick, MA, USA) image processing code Morphometrics (63) was used to segment cells and to identify cell outlines from phase-contrast or fluorescence microscopy images. For time-lapse imaging, raw images were first segmented with the machine learning algorithm DeepCell (64) and then processed with Morphometrics to obtain cell contours. A local coordinate system was generated for each cell outline using a meshing method adapted from MicrobeTracker (65). Cell widths were calculated by averaging the distances between contour points perpendicular to the cell midline, excluding contour points within the poles and sites of septation. Cell length was calculated as the length of the midline from pole to pole. The meshing method failed on cells that lose rod shape. Therefore, for the *mreB* data in Fig. 5E and F and Fig. S5H, length and width were defined as the dimensions of the major and minor principal axes of the cell contour.

For each strain, average cellular dimensions were estimated by first eliminating all cells with widths of <0.6 μm or >2.0 μm (except for the *mreB* data in Fig. 5 and Fig. S5, where no width filtering was performed) and then, if at least 100 cells remained, calculating the median cell length and width. Coefficients of variation (CV) were estimated using the median absolute distance multiplied by 1.4826 to robustly estimate the standard deviation.

To correct for plate effects caused by imaging uninduced cells in 96-well plates rather than in 384-well plates, each of the seven 96-well plates was normalized by subtracting the median length and width of all strains on the plate from the values of each strain and then adding back the global median length and width.

### Single-cell time-lapse imaging.

Overnight cultures were diluted 1:100 and placed onto 1% agarose pads containing LB. For imaging experiments with log-phase cells, overnight cultures were diluted 1:200 into a test tube and grown for 2 h prior to imaging. Phase-contrast images and epifluorescence images (for reporter strains) were acquired with a Nikon Ti-E inverted microscope (Nikon Instruments) using a 100X (numerical aperture [NA], 1.40) oil immersion objective and a Neo 5.5 sCMOS camera (Andor Technology). The microscope was outfitted with an active-control environmental chamber for temperature regulation (HaisonTech, Taipei, Taiwan). Images were acquired using μManager v.1.4 (66).

### Microfluidics.

Overnight cultures were diluted 1:200 and incubated at 37°C for 2 h with shaking. Cells were then loaded into B04A microfluidic perfusion plates (CellASIC). Imaging was performed with a constant flow of fresh LB medium with IPTG at 2 lb/in^2^.

### Transcriptional reporter plasmid construction and quantification.

We used transcriptional reporter plasmids selected from, or designed to mimic, a previously described library of reporter plasmids (27). If the desired reporter was not a member of the library, the upstream region (150 to 400 bp upstream of the ORF and 50 to 100 bp within the ORF and containing the targeted protospacer) was amplified by PCR (Table S1) from BW25113 genomic DNA with 25 bp of flanking sequence and assembled by HiFi (New England Biolabs number E2621L) with the PCR-amplified pUA66 vector. In the case of feedback reporters, PAM mutations were introduced by quick-change mutagenesis (Table S1). Plasmids were transformed into CRISPRi strains by electroporation, selecting for kanamycin resistance. GFP fluorescence was quantified by summing the intensity values of each pixel within the cell contour after subtracting background fluorescence and normalized to the projected area of the cell.

### RT-qPCR experiment design and analysis.

E. coli CRISPRi strains were grown in triplicate from single colonies in prewarmed 4 ml LB for 2.5 h before back-dilution (1:80) in prewarmed 4 ml LB with or without 1 mM IPTG and growth for 3 h prior to collection (OD_600_ ∼0.2). The control strains express *rfp* with or without an sgRNA targeting *rfp* (“nontargeting”) and were treated identically. Samples were collected (300 μl) in 900 μl TRIzol-LS (Thermo Fisher number 10296010) and stored at −20°C overnight. The following day, RNA was extracted according to the TRIzol protocol. RNA was quantified using a NanoDrop 2000c spectrophotometer (Thermo Scientific) to normalize input (500 ng input per 20-μl reaction). For each RT-qPCR probe set and each sample replicate, reactions were performed in triplicate. All RT-qPCR assays were performed using the Luna Universal one-step RT-qPCR kit (New England Biolabs number E3005S) according to its RT and cycling protocols in 96-well PCR plates (Neptune number 3732.X) and measured on a CFX Connect real-time system (Bio-Rad).

Standard curves for each primer pair were first assessed on serially diluted RNA extracted from the CRISPRi control strain to confirm single melting peaks and strong correlations of technical replicates and to calculate their efficiencies in accordance with reference 67. Measured primer efficiencies were used to calculate the relative expression of each gene of interest in each experimental sample relative to the reference gene (*recA*).

### Statistical methods.

Unless otherwise described, robust statistics were used. The mean was estimated using the median, the standard deviation was estimated by multiplying the median absolute distance by 1.4826, and all linear regressions were performed using MM-estimation as implemented in the robustbase R package.

### Strain availability.

The sgRNA library, which has been single colony purified and sequence verified, will be available for distribution through the Coli Genetic Stock Center (CGSC). Mating these strains with the pseudo-Hfr *dcas9* strain (also available from CGSC) or otherwise introducing dCas9 (e.g., on a plasmid) will allow researchers to quickly generate a CRISPRi strain of interest.

### Data availability.

All data referenced are available in the supplemental materials. All raw sequencing data used to calculate relative fitness have been deposited in the Short Read Archive under accession number PRJNA669343. All raw sequencing data used to determine strain purity have been deposited in the Short Read Archive under accession number PRJNA728203.
