# Supplementary figures and images for: Morphological and Transcriptional Responses to CRISPRi Knockdown of Essential Genes in Escherichia coli
Source: mBio. 2021 Oct 12;12(5):e02561-21. doi: 10.1128/mBio.02561-21 (PMC8510551; doi:10.1128/mBio.02561-21)

A

sgRNA strains

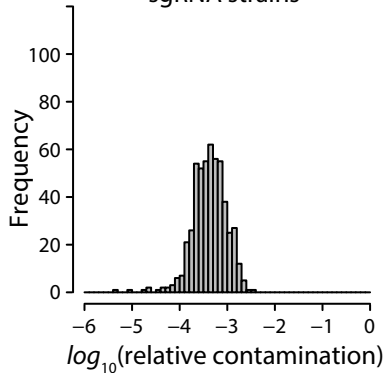

B

CRISPRi strains

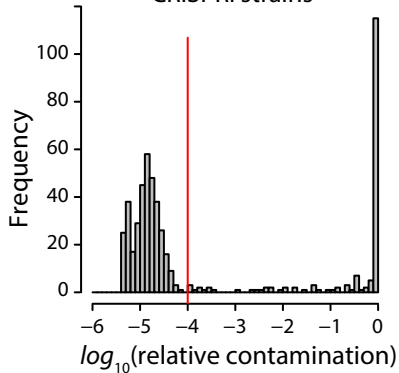

Supplement: FIG S1 [file mbio.02561-21-sf001.pdf]

A

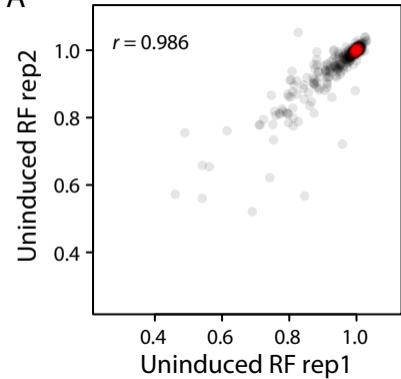

B

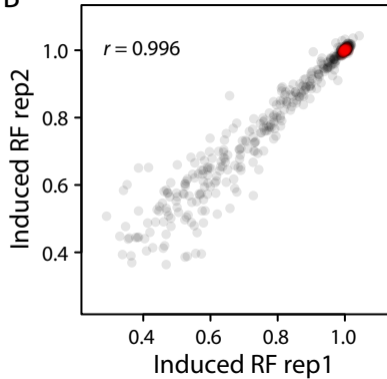

Supplement: FIG S2 [file mbio.02561-21-sf002.pdf]

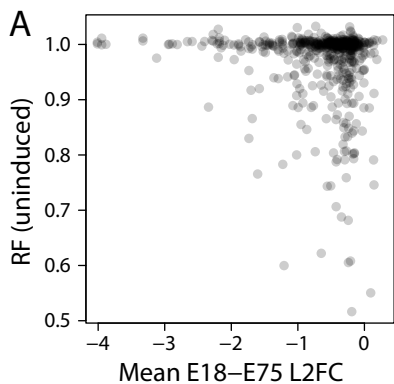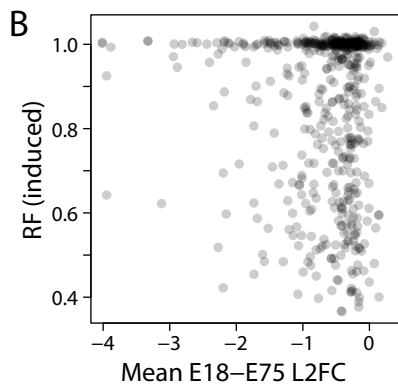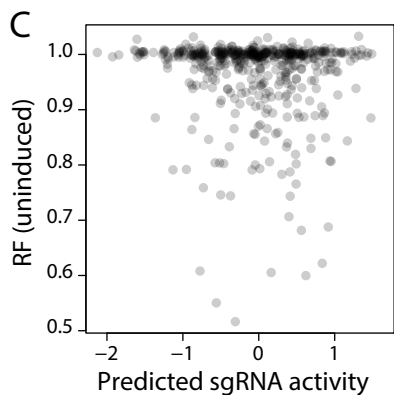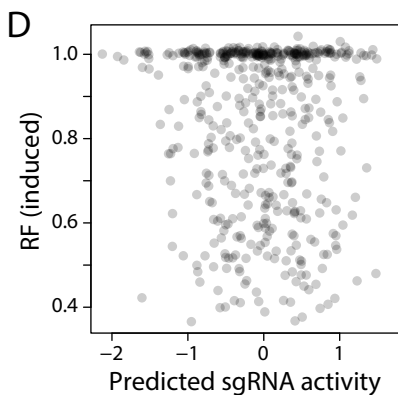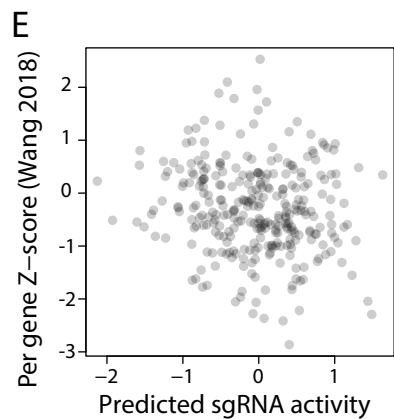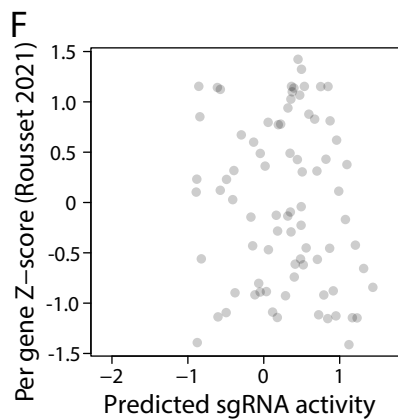

Supplement: FIG S3 [file mbio.02561-21-sf003.pdf]

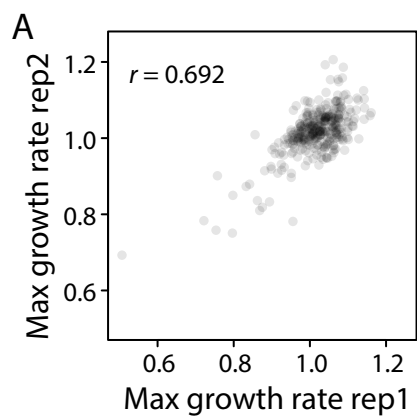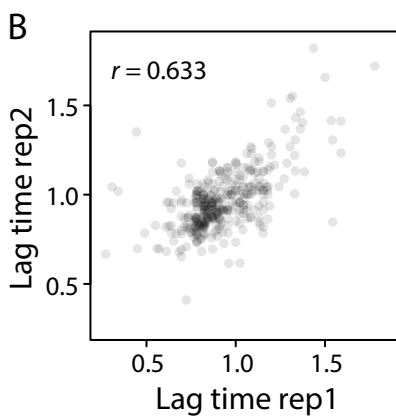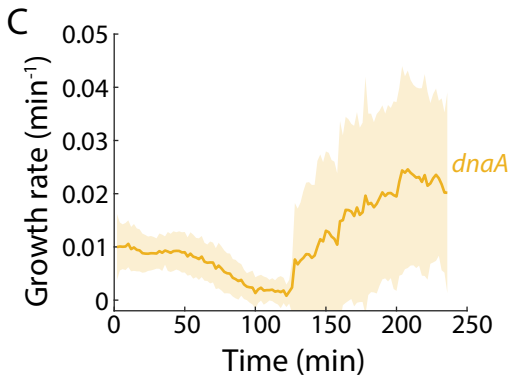

Supplement: FIG S4 [file mbio.02561-21-sf004.pdf]

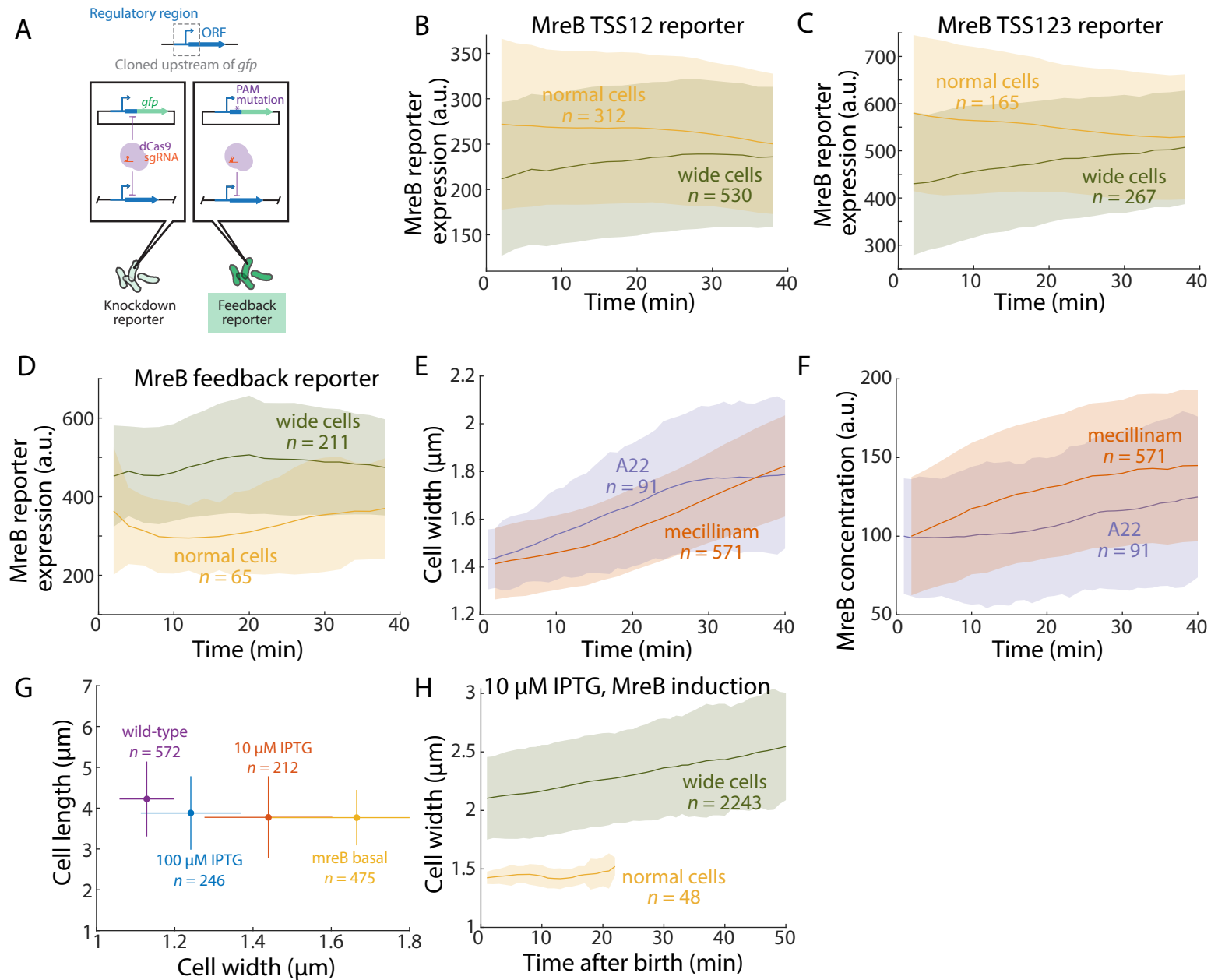

Supplement: FIG S5 [file mbio.02561-21-sf005.pdf]
